# Polymer-Based Gas Sensors for Detection of Disease Biomarkers in Exhaled Breath

**DOI:** 10.3390/bios16010007

**Published:** 2025-12-22

**Authors:** Guangjie Shao, Yanjie Wang, Zhiqiang Lan, Jie Wang, Jian He, Xiujian Chou, Kun Zhu, Yong Zhou

**Affiliations:** 1 School of Future Science and Engineering, Soochow University, Suzhou 215222, China; 2 Science and Technology on Electronic Test and Measurement Laboratory, School of Instrument and Electronics, North University of China, Taiyuan 030051, China; 3 Key Laboratory of Optoelectronic Technology and Systems of Ministry of Education of China, Chongqing University, Chongqing 400044, China

**Keywords:** conductive polymers, gas sensors, exhaled breath, biomarker

## Abstract

Exhaled breath analysis has gained considerable interest as a noninvasive diagnostic tool capable of detecting volatile organic compounds (VOCs) and inorganic gases that serve as biomarkers for various diseases. Polymer-based gas sensors have garnered significant attention due to their high sensitivity, room-temperature operation, excellent flexibility, and tunable chemical properties. This review comprehensively summarized recent advancements in polymer-based gas sensors for the detection of disease biomarkers in exhaled breath. The gas-sensing mechanism of polymers, along with novel gas-sensitive materials such as conductive polymers, polymer composites, and functionalized polymers was examined in detail. Moreover, key applications in diagnosing diseases, including asthma, chronic kidney disease, lung cancer, and diabetes, were highlighted through detecting specific biomarkers. Furthermore, current challenges related to sensor selectivity, stability, and interference from environmental humidity were discussed, and potential solutions were proposed. Future perspectives were offered on the development of next-generation polymer-based sensors, including the integration of machine learning for data analysis and the design of electronic-nose (e-nose) sensor arrays.

## 1. Introduction

Breath analysis has emerged as a powerful and noninvasive diagnostic approach for assessing physiological and pathological states through the detection of volatile organic compounds (VOCs) and inorganic gases exhaled from the human body [1,2,3,4,5]. Exhaled breath contains more than three thousand identifiable gases originating from endogenous metabolic processes, inflammatory responses, and environmental exposures [6,7,8]. Many of these exhaled gases have been recognized as reliable biomarkers for specific diseases—for instance, ammonia (NH_3_) for renal dysfunction, hydrogen sulfide (H_2_S) for hepatic impairment and oral malodor, acetone for diabetes, and nitrogen dioxide (NO_2_) for respiratory inflammation [9,10,11,12]. Compared with conventional invasive diagnostic techniques such as blood or tissue sampling, breath analysis offers significant advantages, including safety, simplicity, rapid response, and the potential for real-time, continuous, and home-based disease monitoring [13,14,15]. Consequently, it has attracted extensive attention across medical diagnostics and public health surveillance [16].

The development of highly sensitive and selective gas sensors is fundamental to realizing accurate breath-based diagnostics [4,17,18]. Among the various gas-sensing technologies (optical, electrochemical, piezoelectric, and field-effect types), chemiresistive gas sensors stand out due to their high sensitivity, simple device architecture, straightforward signal transduction, high compatibility with integrated electronics, and scalability toward miniaturized or flexible systems [19,20]. In particular, conducting polymer-based resistive sensors represents an important subcategory that combines the advantages of high sensitivity, low power consumption, and room-temperature operation [21]. Typical conducting polymers such as polyaniline (PANI), polypyrrole (PPy), and polythiophene (PTh), as well as their hybrid composites with metal oxides, carbon nanomaterials, and two-dimensional (2D) materials, exhibit tunable electrical conductivity and rich chemical reactivity that can be tailored through molecular design, doping engineering, and surface functionalization [22,23]. These features endow polymer-based sensors with superior performance for detecting trace-level disease biomarkers in exhaled breath [24,25].

Compared with conventional metal-oxide semiconductor (MOS) sensors, which need elevated operating temperatures exceeding 200 °C, polymer-based sensors operate efficiently at or near room temperature [8,15,26]. This characteristic not only minimizes energy consumption but also facilitates their integration into flexible, wearable, and portable diagnostic platforms. Moreover, the chemical versatility of polymers enables the introduction of functional groups or dopants that confer selective adsorption toward specific analytes, while composite architectures incorporating hierarchical nanostructures can further enhance charge-transfer efficiency and diffusion kinetics [27]. Through the rational combination of interface engineering, hierarchical porous structuring, and functional nanofillers, recent studies have pushed the detection limits of polymer-based sensors into the sub-ppm and even ppb ranges. These advancements have greatly expanded their applicability to early-stage disease diagnosis by enabling accurate and reproducible detection of exhaled biomarkers.

Despite these advances, several intrinsic challenges remain unresolved. Conducting polymers are prone to signal drift, humidity interference, and chemical instability, all of which can degrade selectivity and reproducibility over long-term operation [20,26]. The nonlinear and multivariate response behavior of polymeric materials also complicates quantitative calibration and cross-sensitivity correction, especially in complex gas mixtures. Overcoming these limitations requires not only innovative material and device engineering but also the integration of data-driven analytical approaches. In this context, machine learning (ML) has recently emerged as a transformative tool for enhancing the performance of polymer-based sensors [4,23,27,28]. By enabling advanced signal processing, pattern recognition, and drift compensation, ML algorithms significantly improve the robustness, precision, and adaptability of gas detection. When integrated into polymer-based electronic-nose (E-nose) systems, these intelligent frameworks facilitate multidimensional feature extraction and self-learning, paving the way for smart, autonomous sensing platforms [4,25].

Despite the rapid expansion of research on gas-sensing materials and electronic-nose technologies, a systematic and domain-specific review focusing on conducting polymer–based sensors for breath-borne disease biomarkers remains limited [13]. Previous reports typically emphasize improvements in materials or device performance. Critical practical challenges, such as humidity interference, signal drift, and cross-sensitivity in complex gas matrices, have not been comprehensively highlighted in the context of real exhaled-breath environments. Furthermore, although machine learning has demonstrated substantial potential in enhancing the robustness and quantitative capability of polymer-based sensors, systematic summarization remains lacking [29].

To address these gaps, this review aims to provide a comprehensive overview of recent progress in polymer-based resistive gas sensors for the detection of disease biomarkers in exhaled breath. Then, this work introduces the fundamental sensing principles, conduction mechanisms, and performance evaluation indicators of resistive polymer-based sensors. Thereafter, the latest developments in materials design and sensing performance for representative biomarkers are discussed, including NH_3_, H_2_S, H_2_, NO_X_, and acetone. In addition, the integration of machine learning algorithms and E-nose architectures for intelligent signal interpretation and system-level optimization is introduced, followed by perspectives on emerging research directions and technological challenges. Collectively, this review aims to elucidate the structure-activity relationships of polymer-based gas sensors and to outline strategies toward next-generation intelligent, stable, and miniaturized platforms for noninvasive disease diagnostics.

## 2. Gas-Sensing Mechanisms and Performance

### 2.1. Principle and Sensing Mechanism

Chemiresistive gas sensors detect gases by measuring changes in electrical resistance upon exposure to target gases [30]. The sensing process can be summarized as follows: gas molecules are first adsorbed onto the surface of the sensitive layer, where interfacial reactions occur, and active sites are occupied. As a result, the carrier concentration or carrier mobility within the sensing layer is modulated, leading to measurable changes in conductivity [31]. After signal conditioning, these resistance changes can be quantitatively related to gas concentration. In essence, resistive sensing relies on the reversible modulation of the electrical properties of the sensing material by gas–solid reactions, thereby mapping gas concentration to resistance variation [32].

As one typical resistive sensor, conducting-polymer gas sensors utilize functional polymers as the active layer to convert gas adsorption events into measurable electrical signals [33]. Their sensing mechanisms primarily involve physisorption, chemisorption, redox reactions, and doping or dedoping processes, which can markedly modulate the carrier density and mobility along the polymer backbone. In conducting polymers, the microscopic structural organization of polymer chains critically determines the accessible pathways, diffusion depth, and overall uptake of gas molecules, thereby fundamentally influencing the sensing response. Polymer chain conformation refers to how the conjugated backbone adopts an extended, coiled, or tightly packed arrangement in three-dimensional space. When the chains are more extended and loosely packed, there is greater interchain spacing and increased fractional free volume, allowing gas molecules to more readily penetrate into interstitial regions and diffuse into the bulk. Conversely, tightly packed conformations restrict interchain distances and reduce accessible free volume, such that gas sorption is largely confined to the surface with limited penetration to deeper active sites [30]. In other words, a more “open” chain conformation translates to increased three-dimensional diffusion space and facilitated initial gas absorption, whereas a tightly coiled chain conformation raises the energetic barriers for diffusion, limiting both the rate and extent of adsorption.

Crystallinity modulates these effects by defining the relative proportions of ordered crystalline domains versus disordered amorphous regions within the polymer matrix. Crystalline domains, characterized by closely packed and highly ordered chain segments, possess minimal free volume and are effectively impermeable to small gas molecules. In contrast, amorphous regions exhibit irregular chain packing with larger free-volume elements, offering the primary diffusion network for gas uptake. Accordingly, polymers with lower overall crystallinity often show enhanced gas absorption because the predominance of amorphous regions facilitates the formation of continuous diffusion channels. High crystallinity, by contrast, suppresses deep diffusion by interrupting these channels, confining adsorption to superficial or localized amorphous pockets. Moreover, the interfaces between crystalline and amorphous phases frequently contain structural defects, microvoids, and gradients in free volume, which can serve as energetically preferential sites for initial gas adsorption, further accentuating the influence of structural heterogeneity [34].

Segmental mobility, defined as the ability of local chain segments to undergo rotational or translational motion, plays a dynamic role in gas absorption kinetics. When segmental mobility is high, chain segments can more easily undergo local rearrangements in response to approaching gas molecules, temporarily enlarging free-volume elements and creating transient diffusion pathways that facilitate deeper and faster gas ingress. This dynamic structural relaxation can also contribute to polymer swelling, increasing overall gas uptake and amplifying perturbations to charge-transport pathways. Conversely, restricted segmental mobility, due to factors such as excessive rigidity of the backbone, strong interchain interactions, or over-doping, hinders such rearrangements, slowing diffusion rates, limiting absorption depth, and lengthening response times. Thus, segmental mobility acts as a “dynamic facilitator” of gas transport and adsorption, and its magnitude directly influences both the kinetics and reversibility of the sensing process [35].

**Figure 1:**
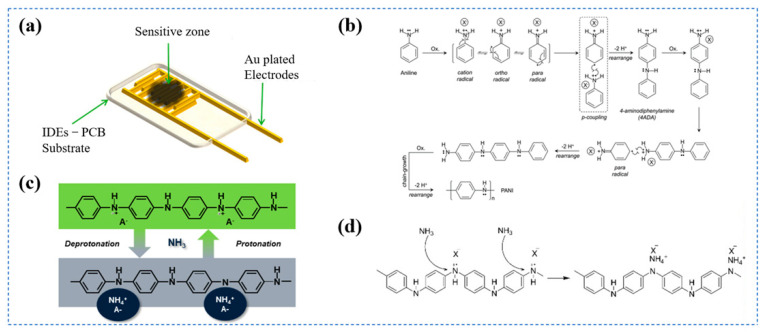
(**a**) A Schematic diagram of a chemiresistive gas sensor, reprinted with permission from Ref. [40]. Copyright 2025, MDPI. (**b**) Chemical structures of some typical conducting polymers, reprinted with permission from Ref. [41]. Copyright 2023, IntechOpen Limited. (**c**) Protonation/deprotonation process in the sensing mechanism of PANI, reprinted with permission from Ref. [42]. Copyright 2025, MDPI. (**d**) “Chemical doping”—redox reaction of PANI, reprinted with permission from Ref. [43]. Copyright 2025, MDPI.

Commonly employed conducting polymers include polyaniline (PANI), polypyrrole (PPy), polythiophene (PTh), and derivatives such as poly(3,4-ethylenedioxythiophene) (PEDOT) [36]. These polymers are typically synthesized via chemical or electrochemical oxidative polymerization [6] and are often patterned into well-defined micro- or nanostructures on interdigitated electrodes to enhance sensitivity [37]. Figure 1 exhibits the typical structure and working mechanism of conducting-polymer gas sensors. As illustrated in Figure 1a, the conductive polymer-based gas sensor consists of interdigitated electrodes coated with a sensitive polymer layer. Figure 1b shows the chemical structures of commonly used conducting polymers, such as PA, PPy, PT, PANI, and PEDOT. The physical and chemical properties of conductive polymers are highly dependent on their doping level. As exhibited in Figure 1c, the acid-doping process in polyaniline would regulate its electrical conductivity [15,38]. Redox reactions modulate the doping level in conducting polymers via electron transfer to or from target gas molecules. This electron transfer process alters key electronic properties of the sensing material, notably its work function and electrical resistance. The doping reactions are typically observed when polymers are exposed to redox-active gases. For instance, electron-accepting analytes such as NO_2_ can withdraw electrons from the conjugated aromatic rings of the polymer backbone. As for p-type conducting polymers (PANI, PPy), the reaction increases both the electrical conductance and the doping level within the polymer, leading to the elevation of resistance. Figure 1d illustrates the interaction between ammonia molecules and polymer chains, highlighting the fundamental sensing mechanism of chemical doping [16]. Compared with conventional metal-oxide sensors, conducting-polymer sensors offer multiple advantages, including room-temperature operation, low power consumption, high sensitivity to trace gases, rapid response, facile fabrication, and mechanical flexibility. In addition, their performance can be further optimized through precise molecular design and structural engineering.

As an essential component of thin-film gas sensors, electrodes function primarily to convert variations in the electrical conductivity of the sensing film into measurable current or voltage signals, thereby enabling the detection of resistance changes within the device [39]. Among various electrode configurations, interdigitated electrodes (IDEs) are the most widely employed in thin-film gas sensors, as illustrated in the schematic. This electrode geometry effectively reduces the initial resistance of the device and improves the efficiency with which electrical signals from the sensing layer are collected. During practical measurements, the conductivity of the sensing material is typically much lower than that of the electrode itself; thus, the resistance contributed by the sensing film directly above the metal electrode can be neglected. The total output resistance of the gas sensor is therefore dominated by the sensing film located between adjacent interdigitated fingers. Consequently, the sensor resistance depends not only on the intrinsic properties of the sensing layer but also on the geometric parameters of the IDE structure. Previous studies have systematically examined the relationship between IDE geometry and the measured conductance of thin-film gas sensors (Equation (1)) [39]:

(1) $$G=\frac{NW}{d}\left(\frac{L}{N}-d\right)\sigma =\left(\frac{L}{d}-N\right)W\sigma$$

where L and W represent the length and width of the interdigitated electrode region, respectively; N denotes the number of electrode fingers; d is the spacing between neighboring electrodes; and σ is the intrinsic conductivity of the sensing film. This relationship suggests that the resistance of a gas sensor (or equivalently, its conductance) can be effectively tuned by increasing the electrode area or reducing the inter-electrode spacing, offering design strategies for optimizing sensor performance.

### 2.2. Performance Evaluation

Regarding resistive gas sensors, the core performance parameters include response, selectivity, response/recovery times, limit of detection (LOD), and stability. These metrics are interrelated and jointly determine the sensors’ applicability and performance limits in practical environments [44,45].

In resistive gas sensors, the gas response (response) quantifies the change in electrical resistance when the sensing material is exposed to a target gas. This metric reflects interfacial adsorption–reaction processes and the associated modulation of near-surface band structure, carrier concentration, and potential-barrier height. Two commonly used definitions of response are exhibited as Equations (2) and (3) [46,47,48,49,50].

(2) $$Response=\{\begin{array}{cc} \frac{R_{air}}{R_{gas}}, & for\;reducing\;gases\\ \frac{R_{gas}}{R_{air}}, & for\;oxidizing\;gases \end{array}$$

(3) $$Response=\frac{\left|R_{gas}-R_{air}\right|}{R_{air}}\times 100\%$$

where $R_{air}$ denotes the steady-state resistance in the reference atmosphere, and $R_{gas}$ represents the steady-state resistance under exposure to the target gas [44]. Furthermore, the sensitivity of the gas sensor can be interpreted as the slope of the response–concentration relationship within the operating range. Greater sensitivity denotes a larger resistance modulation per unit concentration, enabling discernible signals at trace levels and manifesting as a lower limit of detection [51,52,53]. It should be noted that the ratio-type response and the relative resistance change describe the same sensing behavior, and the two forms of response expression can be mutually converted. For sensors with a relatively small change in resistance after contacting the target gas, the response value is generally defined by the relative change in resistance. For sensors with a relatively large change in resistance after contacting the target gas, the response is typically defined by the ratio of resistances.

Selectivity denotes the ability of a sensor to distinguish the target analyte from interfering species in complex gas mixtures. An ideal selective sensor exhibits a strong response to the target analyte while demonstrating negligible cross-sensitivity to interfering gases. Quantitatively, selectivity is often assessed by the ratio of the response to the target gas over the responses to interfering gases, and a high ratio indicates superior discriminative capability [54,55].

As for the gas sensor, the response time serves as a metric for the detection speed of the device, whereas the recovery time characterizes its efficiency in purging residual analytes and restoring baseline sensor functionality [46]. The response time is defined as the duration required for the sensor resistance to reach 90% of its total change upon exposure to the target analyte [51], whereas the recovery time denotes the time needed for the resistance to return to 90% of its baseline value after the removal of the analyte [56,57,58].

The limit of detection (LOD) is the minimum concentration of the target gas that can be reliably distinguished from baseline noise, and thus quantified the sensors’ capability for trace detection [59]. Conventionally, the LOD is defined under a specified signal-to-noise ratio $\left(S/N=3\right)$ [60]. For resistive gas sensors, LOD is typically estimated by extrapolating the response–concentration calibration curve and incorporating the standard deviation of baseline resistance noise, as shown in Equation (4).

(4) $$LOD=\frac{3\sigma }{S}$$

where σ denotes the standard deviation of the baseline noise, and S denotes the sensitivity. A smaller LOD indicates superior performance for ultra-low-concentration detection.

Stability refers to the ability of a sensor to maintain its baseline resistance and response magnitude over prolonged operation and under variations in external conditions [61]. Ideally, the sensor should exhibit minimal baseline drift and no significant loss of sensitivity after multiple exposure–recovery cycles, under changes in ambient temperature and humidity, and during extended continuous operation [62]. Insufficient stability typically manifests as baseline drift and sensitivity degradation, thereby compromising measurement accuracy and reproducibility.

Interface engineering and functionalization of sensing materials are effective approaches to improving the gas-sensing properties of conducting polymers. In particular, to quantitatively assess the regulatory influence of functional modifications on the sensing performance of conducting-polymer active layers, the ratio between the response value S_F_ of the functionally modified sensor and the response value S_N_ of its unmodified counterpart (S_F_/S_N_) was introduced.

## 3. Gas Sensors Based on Conducting Polymers

Exhaled breath analysis demonstrates significant potential for early disease screening and diagnosis, as a noninvasive and readily repeatable detection methodology. Human exhaled breath contains a variety of volatile biomarkers, such as ammonia (NH_3_), hydrogen sulfide (H_2_S), hydrogen (H_2_), nitrogen oxides (NO_X_), and acetone, whose concentration changes are closely associated with specific pathological conditions, as shown in Table 1. For instance, acetone in exhaled breath has been extensively studied as a potential biomarker for diabetes mellitus and lung cancer. To present a clearer overview of the associations between these gaseous biomarkers and corresponding diseases, along with their reference concentration ranges and research basis, the key information is summarized in the table below.

### 3.1. NH_3_

**Figure 2:**
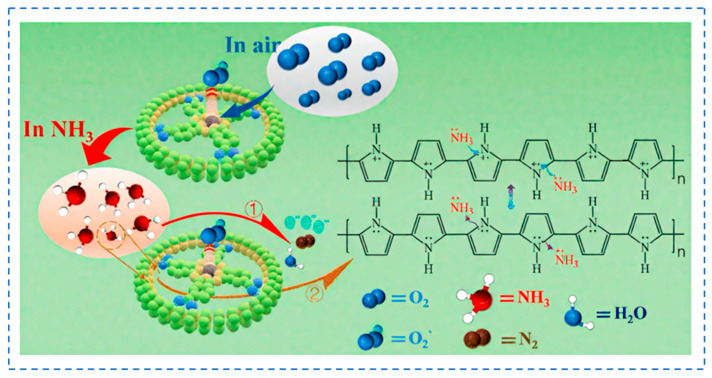
Schematic diagram of the resistive gas-sensing mechanism of conducting polymers (typically polyaniline, PANI) toward NH_3_, reprinted with permission from Ref. [13]. Copyright 2025, MDPI.

NH_3_ is a strongly basic, irritating, and highly water-soluble inorganic volatile gas with significant monitoring relevance across environmental management, livestock and occupational health, and noninvasive medical diagnostics. Clinically, exhaled NH_3_ is closely associated with nitrogen metabolism and renal function. For instance, the average concentration of ammonia in the exhaled breath of patients with chronic kidney disease (CKD) ranges from 153 ppb to 130,000 ppb, which significantly exceeds that observed in healthy populations [67]. PANI is a typical sensitive material toward NH_3_. When PANI was exposed to NH_3_, the NH_3_ molecules captured holes from =NH^+^- and -NH_2_^+^- groups of PANI and caused it to transfer from emeraldine salt (ES) form to emeraldine base (EB) form, leading to a decreased conductivity of the PANI [68] (Figure 2). However, pure PANI suffers from poor long-term stability, sluggish response/recovery times, and low sensitivity. To enhance the NH3-sensing performance of conductive polymers, various interface engineering and functionalization methods were proposed [69].

Ma et al. synthesized a hierarchical 0D/2D/3D MXene@Zn_3_In_2_S_6_@PANI heterostructure through a multistep strategy comprising template-assisted formation of hollow MXene microspheres, hydrothermal growth of Zn_3_In_2_S_6_ nanosheets, and ice-bath-assisted in situ polymerization of PANI nanoparticles [70]. The resulting architecture, characterized by uniformly distributed 0D PANI on 2D Zn_3_In_2_S_6_ supported by a 3D MXene framework, generated rich heterointerfaces and facilitated gas diffusion and charge transport. Benefiting from this structural design, the optimized MXene@Zn_3_In_2_S_6_@PANI sensor exhibited a response of 484.28% toward 100 ppm NH_3_ at room temperature and 45% RH, which was approximately 2.39 times higher than that of pristine PANI. In addition, the composite gas sensors achieved 1183.24% under 90% RH, together with a detection limit of 573.105 ppb, rapid response/recovery (200.2/95.7 s), excellent selectivity, strong humidity tolerance, and stable 21-day operation (88.52% retention). The superior performance could be attributed to the unique hollow flower-bulb architecture and abundant heterojunctions formed at the composite interface. Nevertheless, the sensor showed incomplete recovery during cyclic testing (78.1% after five cycles), and prolonged high-humidity exposure induced a transient water layer on the PANI surface, partially hindering NH_3_ adsorption at active sites. Abdollahi-Esfahlani et al. fabricated a polyvinyl alcohol/chitosan/polyaniline (PVA/CS/PANI) nanofiber membrane sensor for NH_3_ detection [71]. The amine functional groups present in the chitosan (CS) backbone facilitated the covalent grafting of PANI chains, leading to the formation of a uniform hierarchical architecture. Under room-temperature conditions, at 38% relative humidity, the sensor exhibited a response of 79.49% to 100 ppm NH_3_ and 31.74% to 50 ppm NH_3_, showing a linear relationship (R^2^ = 0.982) within the concentration range of 25–100 ppm. The detection limit was determined to be 571 ppb, with response and recovery times of 150 s and 18 s, respectively. The excellent performance could be attributed to the porous structure and the adsorption effect of chitosan to NH_3_. Furthermore, fluctuations in environmental humidity were found to influence the protonation state of PANI, thereby affecting the accuracy of NH_3_ detection. To promote the humidity-resistant performance of PANI, Liu et al. fabricated a flexible PANI–CeO_2_ composite thin-film NH_3_ sensor based on a polyimide substrate [68]. Aniline monomers and CeO_2_ nanoparticles were in situ polymerized at 10 °C to form a uniform composite sensing film. The composite sensor exhibited response values ranging from 106.9% to 262.7% toward 10–50 ppm NH_3_ at room temperature, which were substantially higher than those of pure PANI. The device was capable of detecting NH_3_ concentrations as low as 16 ppb, with a theoretical limit of detection (LOD) of 0.274 ppb, and displayed a markedly shorter recovery time compared with pristine PANI. The PANI–CeO_2_ sensor demonstrated excellent selectivity, with responses to interfering gases such as formaldehyde HCHO and hydrogen sulfide less than one-tenth of that to NH_3_. Long-term stability tests indicated that the response to 50 ppm NH_3_ remained at 98.2% of its initial value after three weeks of continuous operation. Furthermore, the device retained stable sensing performance after 500 bending and stretching cycles, confirming its mechanical flexibility and potential for integration into flexible electronic platforms. In addition, the PANI–CeO_2_ sensor exhibited the same dynamic response behaviors at 25–75%RH, and a slight reduction in response under 90% RH. The excellent humidity-independent performance benefited from the nice hydrophobicity of CeO_2_. Li et al. developed a hierarchical multifunctional sensor based on industrial-grade thermoplastic polyurethane (TPU) [72]. Polypyrrole (PPy) coatings were subsequently fabricated via the in situ oxidative polymerization of pyrrole using FeCl_3_ as the initiator, carried out at room temperature for 60 min on the TPU substrate. During gas-sensing evaluation, the PPy-modified sensor exhibited a highly linear and concentration-dependent response toward NH_3_ within the range of 10–400 ppm (R^2^ = 0.995). The device also demonstrated rapid response and recovery characteristics, with corresponding times of 23 s and 43 s, respectively, highlighting its capability for fast and quantitative detection in practical environments. In addition, the engineered micropillar array coated with the polymerized PPy layer showed pronounced surface hydrophobicity, characterized by high water contact angles (≥102°). This structural feature is indicative of excellent tolerance to humid conditions and effective suppression of moisture-induced signal interference, thereby contributing to superior operational stability and environmental robustness. Wongrat et al. developed an HCl-doped ZnO/PANI nanocomposite as the sensing material [73]. The ZnO nanostructure was initially synthesized using a direct-current heating method, which promoted uniform crystal growth and ensured high surface activity. Subsequently, in situ oxidative polymerization was employed to introduce polyaniline (PANI) onto the ZnO framework, thereby forming a well-integrated hybrid composite with enhanced gas-adsorption affinity and charge-transfer capability. Under ambient conditions, the resulting sensor delivered an exceptionally high response of 5990 toward 500 ppm NH_3_ and maintained a detectable response level of 5.5 even at concentrations as low as 100 ppb, reflecting its remarkable sensitivity over a wide dynamic range. Furthermore, the device exhibited outstanding gas selectivity and sustained signal stability during a long-term operation period of 40 days, confirming its structural and electrical robustness. These sensing characteristics collectively indicate strong potential of the ZnO/PANI composite sensor for noninvasive breath analysis and early screening of chronic kidney disease through trace NH_3_ detection in human exhaled breath.

Deng et al. proposed a bioinspired s-PANI@PDMS flexible sensing film featuring anisotropic microgroove architectures [74]. The engineered microgrooved surface exhibited pronounced hydrophobicity, with a water contact angle of ~120°, which effectively increased the accessible sensing surface area and optimized gas diffusion pathways. This structural design also enabled stable sensing performance under high-humidity conditions, maintaining reliable operation at relative humidity levels up to 80% RH. Dynamic sensing measurements demonstrated a low detection limit of 0.5 ppm for NH_3_, while a response value of 4.02 was achieved at 60 ppm NH_3_ at room temperature, accompanied by a response time and recovery time of 43 s and 53 s, respectively. In addition, clinical validation further demonstrated that the sensor could effectively discriminate patients with chronic kidney disease (CKD) with varying disease severities based on exhaled NH_3_ concentrations, showing a strong correlation with serum creatinine levels. Although prolonged operation, beyond 28 days, led to gradual deprotonation of PANI and a concomitant decline in sensing sensitivity, the original performance could be fully recovered through HCl vapor re-doping, indicating favorable reusability and long-term maintenance potential. Lee et al. fabricated an NH_3_ sensor based on urchin-like polypyrrole (U_PPy) nanoparticles via dual-nozzle electrospray and vapor deposition polymerization (VDP) [75]. The chemiresistive sensor exhibited a LOD of 0.01 ppm for NH_3_ at room temperature, 10–100 times higher sensitivity than pristine PPy sensors. With response time <1 s and ~30 s recovery for 0.1 ppm NH_3_, it efficiently detects NH_3_, methanol, and acetone, with optimal NH_3_ selectivity and stable response over 100 cycles (attributed to U_PPy’s high specific surface area).

Husain et al. synthesized polythiophene/single-walled carbon nanotube (PTh/SWCNT) nanocomposites via in situ chemical oxidative polymerization [76]. The optimal composite, PTh/SWCNT-3 with 15 wt% SWCNTs, demonstrated an initial electrical conductivity of 6.93715 S/cm at room temperature. It exhibited a significant conductivity response of 3.65 S/cm to 2000 ppm NH_3_, with response and recovery times of 60 s; saturation times at high concentration were 200 s and 180 s. The sensor showed a low detection limit of 5 ppm for NH_3_ and superior selectivity over eight other VOCs compared to pure PTh, along with thermal stability up to 130 °C. Limitations include the lack of investigation into practical factors, the absence of long-term stability data at 5 ppm NH_3_, and unaddressed scalability feasibility.

As a strongly basic gas, NH_3_ can initiate irreversible or semi-irreversible chemical modifications on polymer chains, particularly under prolonged exposure to high concentrations (the 130,000 ppb NH_3_ in the exhaled breath of severe CKD patients). The gas sensors based on PANI would exhibit incomplete recovery and significant baseline drift [70]. In addition, under prolonged or high-concentration exposure, incomplete desorption and the gradual degradation of the polymer structure would affect the stability of the sensor. Some NH_3_ molecules may strongly adsorb onto active sites or diffuse deep into the bulk of the polymer, making them difficult to desorb with some irreversible side reactions. Additionally, the ambient humidity significantly influences the NH_3_ sensing performance of conducting polymers, whereas this effect is complex and non-uniform. The sensor response can increase, decrease, or even peak at an optimal humidity level depending on the specific system. Therefore, strategies to mitigate humidity interference or even exploit it to enhance sensor performance remain a critical area for further investigation.

### 3.2. H_2_S

H_2_S is a highly toxic, strongly reducing, and irritating gas characterized by an extremely low odor-detection threshold [77,78]. At elevated concentrations, olfactory fatigue can occur, leading to underestimation of exposure. Moreover, H_2_S was regarded as the biomarker of asthma, halitosis, and chronic obstructive pulmonary disease [79]. For instance, the exhaled breath of halitosis patients contains a higher level of H_2_S (0.1–0.5 ppm) than that of healthy people [80]. 

Regarding the typical H_2_S gas sensor based on PANI, exposure to H_2_S leads to the protonation of a fraction of the PANI structure, resulting in a measurable decrease in its electrical resistance ($H_{2}S+\mathrm{PANI}↔{\mathrm{HS}}^{-}+{\mathrm{PANIH}}^{+}$). This protonation process enhances the material′s conductivity. However, the sensing performance is inherently limited because the number of active sites within the PANI matrix available for protonation by H_2_S constitutes only a relatively small fraction. The presence of insulating structural domains that remain unaffected by the dopant further restricts the dynamic range of the resistance change, leading to a constrained sensing response magnitude. Hence, the incorporation of nanosensing materials into conductive polymer has become an effective method to overcome these barriers.

**Figure 3:**
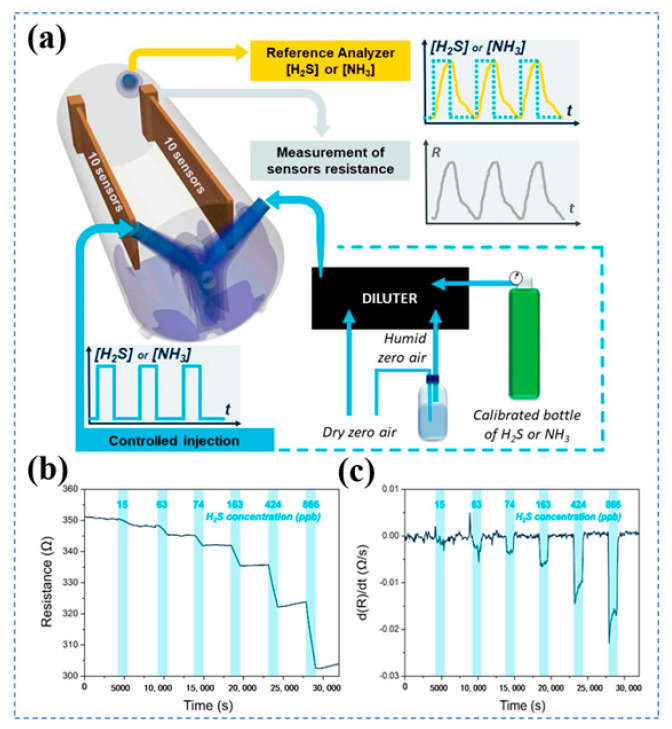
(**a**) The dynamic gas flow system employed for performance evaluation. This setup precisely controls the concentrations of the target gas (H_2_S) and interfering gas (NH_3_), as well as the environmental humidity, while synchronously recording the sensor resistance and reference gas levels. (**b**) Step response plot. (**c**) Derivative response plot, reprinted with permission from Ref. [81]. Copyright 2025, MDPI.

Duc et al. fabricated hydrogen sulfide (H_2_S) sensors using PANI, tin chloride, and PEDOT:PSS as raw materials [81] (Figure 3a). After dispersing PANI in dimethylformamide (DMF), SnCl_2_ was added, followed by compounding with ethylene glycol-containing PEDOT:PSS. The mixture was ultrasonically homogenized, coated onto interdigitated electrodes, and vacuum-dried to obtain the sensors. At room temperature, the sensor exhibited a low detection limit of 28–82 ppb for sub-ppm H_2_S, with a linear response in the concentration range of 50–600 ppb and a response time of less than 100 s. Figure 3b shows that the resistance of the sensor gradually decreased with the gradient increase of H_2_S concentration (15–886 ppb), which directly verified its capability to continuously quantify varying H_2_S concentrations. Figure 3c further characterizes the rapid response/recovery behavior. It also possessed excellent repeatability, reproducibility, and stability over one month. However, humidity significantly affected its sensitivity, and the sensor became more sensitive to NH_3_ after aging, thus requiring humidity control and interference compensation.

Zhang et al. developed a flexible SnO_2_/rGO/PANI ternary composite sensor [82] for H_2_S detection via a multistep synthesis: under an ice bath, aniline monomers were in situ polymerized with rGO and SnO_2_ hollow spheres to form the sensing material. This sensor exhibited a response of 23.9 to 200 ppb H_2_S and a 50 ppb limit of detection (LOD), showing strong trace H_2_S sensing performance. Further tests revealed rapid response/recovery for 2 ppm and 5 ppm H_2_S, with response/recovery times of 82/78 s and 80/88 s, respectively. Enhanced H_2_S sensing stems from synergistic effects at the ternary heterojunctions: the p-n heterojunction (formed by p-type PANI and n-type SnO_2_) acted as a signal amplifier, enabling sub-ppb-level sensitivity for trace H_2_S. Additionally, the sensors were integrated into an array for H_2_S detection in simulated halitosis exhaled breath. The excellent selectivity allowed effective distinction between halitosis patients and healthy individuals.

Bai et al. reported one highly sensitive H_2_S sensor based on polythiophene-WO_3_ composites [83]. As for the synthesis of composites, the WO_3_ was dispersed in chloroform and hybridized with thiophene monomers using FeCl_3_ as an oxidant (molar ratio 3:1) through in situ oxidative polymerization at 0 °C to obtain polythiophene-WO_3_ (PT-WO) composites. Among these, the 10% PT-WO hybrid exhibited the highest performance, with a response of 7 toward 100 ppm H_2_S at 70 °C, approximately 2–3 times that of pure WO_3_, a recovery time under 15 s, and an LOD of 2 ppm. The sensor displayed high selectivity, with selectivity coefficients exceeding 5 for methanol, ethanol, and other interfering gases. The promotion could be attributed to the highly sensitive modification of conductive PT for H_2_S adsorption. Bibi et al. proposed a highly sensitive H_2_S sensor based on carbon aerogel (CA)-PANI composites. The composite sensor showed a high response of 452% toward 50 ppm H_2_S, which was two times higher than that of the PANI sensor. In addition, the CA-PANI sensor exhibited a rapid response time of 1 s, resulting from its porous structure and larger specific surface area. However, due to the adsorption of sufficient H_2_S gas molecules, the dedoping speed of CA-PANI sensor was much slower than that of the pure PANI sensor [84,85]. Saravanan et al. further developed a ternary PANI/SnO_2_/rGO composite H_2_S sensor by a facile one-step hydrothermal approach followed by a polymerization method [86]. The composite exhibited a 56% response to 100 ppm H_2_S with short response/recovery times of 35/40 s, showing strong selectivity toward H_2_S over ethanol and methanol. The sensor maintained 95% of its initial response after 100 days, indicating excellent long-term stability. The excellent performance resulted from the large BET surface area and smaller mesoporous channels in the composites. 

As shown above, H_2_S could interact with conductive polymer via the doping mechanism. On the other hand, as a weak acid, H_2_S is partially doped into the conductive polymers, leading to a low sensitivity. Hence, the development of H_2_S sensors based on conductive polymers poses greater challenges compared to that of NH_3_ sensors. Moreover, the sensing behavior of H_2_S is strongly modulated by environmental moisture, which governs its chemical interaction with the sensing material [87]. Specifically, H_2_S could exhibit reducing behavior in dry, inert atmospheres, where it acts primarily as a reducing agent. In contrast, under humid conditions, the presence of water vapor promotes the acidic character of H_2_S, leading to a protonation-dominated reaction pathway. Hence, environmental humidity would affect the sensing performance of conductive polymer-based H_2_S sensors.

### 3.3. H_2_

H_2_ is an odorless, low-molecular-weight, highly diffusive flammable gas with very low ignition energy and a wide flammability range [88]. Elevated H_2_ levels in exhaled breath originate from the bacterial fermentation of carbohydrates in the intestine, a process often exacerbated by underlying gastrointestinal diseases. In patients with such disorders, microbial dysbiosis can lead to more aggressive fermentation, resulting in the premature and excessive production of H_2_ [89]. 

**Figure 4:**
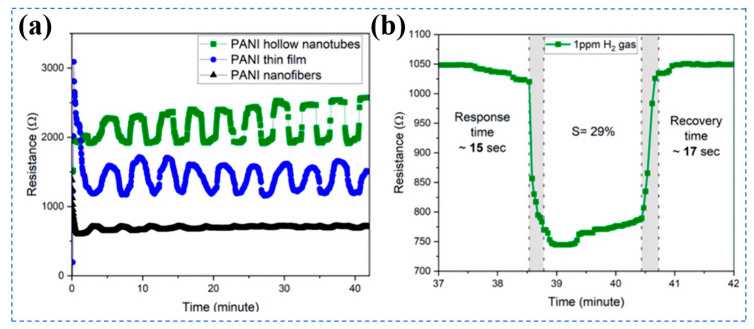
(**a**) Comparison of responses in different PANi at 1 ppm H_2_ detection. (**b**) Response time of hollow PANi nanotube sensor at 1 ppm H_2_ gas, reprinted with permission from Ref. [13]. Copyright 2025, MDPI.

Pure conductive polymer H_2_ sensors suffer from intrinsic limitations, including low intrinsic conductivity, poor processability, and limited solubility, which restrict their large-scale practical applications [90]. To address these challenges, researchers have adopted composite modification strategies for performance optimization. Askar et al. reported a highly sensitive H_2_ sensor based on nanostructured PANI [91]. Room-temperature tests in a nitrogen atmosphere revealed that the PANI hollow nanotubes exhibited a superior sensing performance than PANI thin film and PANI nanofibers, including a high response value of 29% to 1 ppm H_2_, with response/recovery times of 15 s/17 s (Figure 4a,b). On the one hand, the porous structure of PANI hollow nanotubes facilitates the adsorption of H_2_. On the other hand, the electrical conductivity (9.15 S/cm) and carrier mobility of PANI hollow nanotubes are significantly higher than those of PANI thin films (0.60 S/cm) and PANI nanofibers (0.05 S/cm), indicating a better connection network for carrier transfer and avoiding the accumulation of electrons at the interface during the gas-solid reaction process.

Sunghun Cho et al. developed a highly sensitive H_2_ sensor based on Pd-decorated nanoporous P(AN-co-ASA):PSS, with detailed synthesis and structure. After copolymerizing aniline and aniline-2-sulfonic acid, abundant -SO_3_H groups from the comonomer and PSS anchored 3–10 nm Pd nanoparticles; meanwhile, ammonium bicarbonate (water-soluble porogen) decomposition created 10–30 nm pores in the material. Under room temperature and simulated air, the sensor exhibited excellent performance: a low LOD of 5 ppm, a rapid response/recovery speed of 90/40 s, outperforming Pd-decorated non-porous and pristine P(AN-co-ASA):PSS structures. Its superior performance was derived from two core designs: (1) -SO_3_H -anchored Pd avoided Pd-nitrogen complexes (which impair charge transport) while enhancing Pd-H_2_ interaction (as per its working mechanism); (2) porogen-constructed nanopores raised the material’s BET specific surface area to 23.1 m^2^/g, accelerating H_2_ diffusion and improving its contact with the sensing matrix. Notably, the sensor showed much higher selectivity for H_2_ over common interferents, highlighting its potential for practical H_2_ detection [92].

As for the sensing mechanism, hydrogen detection involves the dissociation of hydrogen molecules and their subsequent binding to nitrogen atoms along the PANI backbone [93]. The interaction leads to an increase of charge carriers (holes), thereby reducing the electrical resistance of the material. The process continues with a charge transfer between adjacent amine nitrogen sites, which restores the polymer to its initial doped, conductive state (emeraldine salt). This reversible redox reaction is the basis for the observed conductivity change. The conductivity of PANI is highly dependent on its doping level, which is altered by the transfer of electrons to or from the gas molecules during this surface interaction. Hydrogen is relatively inert and lacks specific and strong interactions with the backbone of conductive polymers, resulting in weak sensitivity. Additionally, humidity can significantly affect sensor performance. On the one hand, water molecules affect the protonation state of polymers. On the other hand, the presence of moisture can interfere with the sensor’s response to hydrogen gas, potentially leading to erroneous readings or reduced sensitivity.

### 3.4. NO_X_

NO_X_ is a strongly oxidizing, irritating gas whose ambient and indoor backgrounds typically fall within the ppb-sub-ppm range [94], while near-source emissions and occupational settings can reach ppm–tens of ppm. Moreover, elevated levels of NO_2_ in exhaled breath condensate (EBC) have been reported in patients with various inflammatory lung diseases, notably bronchial asthma, bronchiectasis, and chronic obstructive pulmonary disease (COPD) [95]. 

For typical conductive polymer PANI, on the one hand, NO_2_ acts as a dopant, and the resistance of PANI continuously decreases upon exposure to NO_2_. On the other hand, NO_2_ is an oxidizing gas (electron acceptor). When it interacts with p-type PANI, it captures electrons from the polymer matrix, leading to a reduction in resistance. Umar et al. engineered NO_2_ sensors based on a polyaniline/silver oxide/graphene oxide (PANI/Ag_2_O/GO) composite [96]. At the optimized operating temperature of 100 °C, the sensor exhibited a response of 5.85 toward 25 ppm NO_2_—significantly higher than that of pristine PANI (2.5) and the binary PANI/Ag_2_O composite (3.25). Additionally, the device showed rapid and stable response characteristics in the 5–50 ppm NO_2_ concentration range, confirming excellent reproducibility and operational stability. The enhanced sensing performance stems from the synergistic heterointerface in the PANI/Ag_2_O/GO composite: improved charge transfer and abundant adsorption sites collectively contribute to its superior sensitivity and selectivity.

Zheng et al. reported a ZnO/PANI nanoflake array sensor for highly sensitive and rapid detection of NO_2_ at room temperature [97]. As for the composite synthesis procedure, ZnO nanoflake arrays were initially synthesized by a hydrothermal method, and then a vapor phase diffusion method was employed to regulate the aniline diffusion time (with 0.5 h as the optimal parameter) for growing a PANI film on the ZnO surface, with the formation of n-p heterojunctions. The ZnO/PANI sensor exhibited a high response of 28.00 towards 10 ppm NO_2_ at room temperature, which was 23.53 times higher than that of pure PANI. Furthermore, the ZnO/PANI showed a limit of detection as low as 0.01 ppm and short response/recovery times of 32/18 s. Moreover, the sensor showed excellent selectivity for NO_2_, with a response of only 1.00–4.66 towards 100 ppm interfering gases. A slight degradation of response toward NO_2_ was observed with the increase in humidity from 10%RH to 70%RH, indicating the remarkable humidity tolerance. The enhanced mechanism could be attributed following factors. The heterojunction interface formed by PANI and ZnO possessed high activity, which would facilitate the adsorption of NO_2_. Additionally, the excellent carrier transport property of PANI would promote the electron migration during the reaction. Kamble et al. proposed a highly efficient room temperature NO_2_ sensor based on interconnected nanofibrous polythiophene (INPTh) [98]. The INPTh sensor achieved 47.58% response at 100 ppm NO_2_ and 2.46% at 1 ppm NO_2_, with rapid response/recovery times of 15/8050 s. Furthermore, the INPTh sensor exhibited nice selectivity toward NO_2_ due to the high electron affinity (289 kJ/mol) of NO_2_, which was larger than that of NH_3_ (225.4 kJ/mol) and H_2_S (55.2 kJ/mol). Zhao et al. constructed TiO_2_-nanotube/PPy/MoO_x_ heterostructures as the ultrahigh-sensitive NO_2_ sensing material at room temperature for asthma diagnosis [99]. The composites were synthesized by integrating MoOx and conductive PPy onto a TiO_2_ nanotube array (TiNT) through direct electropolymerization. Due to the as-formed double p-n heterojunctions (TiO_2_/PPy and PPy/MoO_x_), the TiO_2_/PPy/MoOx sensor exhibited remarkable sensing performance to NO_2_, including a high response of 11.96 to 1 ppm NO_2_, short response/recovery times of 9/11 s, and excellent selectivity. Resulting from the Mo-N coupling, the carrier transfer across the PPy/MoO_X_ interface was significantly promoted, thus contributing to the high response and rapid kinetics. Moreover, the as-prepared sensor could accurately detect NO_2_ levels in human exhaled breath. In addition to the above materials engineering, the deposition and structure of the polymer film also affect the sensing performance of the gas sensor. Xie et al. compared the sensing performance of Langmuir-Blodgett films of neat polyaniline and anthranilic-acid-doped polyaniline with self-assembled PANI–PSSA multilayers [100]. At 20 ppm NO_2_, trilayer Langmuir-Blodgett films responded in about 10 s and recovered in roughly 4 min, whereas bilayer self-assembled films responded in 8 s and recovered in 2 min. The results exhibited that the thinner the films, the higher the sensitivity and faster response speed would be achieved.

S.T. Navale et al. synthesized PTh via chemical oxidative polymerization with thiophene as the monomer and FeCl_3_ as the oxidant. The PTh was dissolved in m-cresol to form a casting solution [101], which was deposited onto glass substrates by spin-coating and dried at 100 °C for 10 min to fabricate PTh thin-film NO_2_ sensors. Room-temperature tests showed the sensor exhibited a 9–33% response toward 10–100 ppm NO_2_, with response increasing monotonically with concentration. Its selectivity coefficient for NO_2_ was 8.25–33, markedly superior to that for interfering gases (NH_3_, H_2_S, C_2_H_5_OH). With a response time of 220–297 s, recovery time of 585–1603 s, and good repeatability, the favorable performance is attributed to the PTh film’s porous microstructure facilitating gas diffusion and interfacial reactions.

Notably, some polymer-based NO_2_ gas sensors exhibited incomplete recovery to their electrical baseline following exposure to gas, even at elevated temperatures of up to 80 °C [102]. This irreversible response severely compromises the fidelity and long-term stability. Additionally, humidity can adversely impact the NO_2_ sensing performance of sensors due to the high solubility of NO_2_ in water. 

### 3.5. Acetone

Acetone is a low-molecular-weight, highly volatile, polar VOC [103]. Ambient and indoor backgrounds are typically at the ppb level, but ppm-level excursions can occur during solvent use, printing/coating, medical and laboratory operations, and in near-source or confined environments [104]. It is found that the acetone concentration in the exhaled breath of healthy people ranges from 300 to 900 ppb [105]. While acetone concentrations are 2.2–22 ppm for type 1 diabetes patients and 1.76–9 ppm for type 2 diabetes patients [106]. Hence, acetone could be seen as a biomarker of diabetes. 

Upon exposure to acetone vapor, acetone molecules are absorbed into the porous surface of the conductive polymer film. A hydrogen bond can form between the carbonyl oxygen (C=O) of acetone and the hydrogen atom of the N-H group within the pyrrole ring. This interaction weakens the C=O bond of acetone due to the partial donation of the lone pair electrons from the oxygen atom, leading to the formation of an N-H···O=C hydrogen bond. The resulting hydrogen-bonding network between the acetone molecules and the repeating units of the conductive polymer chain can impede intra-chain and inter-chain electron hopping. This disruption in charge-transport pathways leads to a decrease in the overall conductivity of the polymer, which is measured as an increase in electrical resistance. Lee et al. prepared PANI/NiO/TiO_2_ composites for highly sensitive detection toward acetone at room temperatures [107]. In addition to the material modification, UV irradiation was also utilized to promote the sensing performance. Under 360 nm ultraviolet irradiation at 2 mW cm^−2^ and 25 °C, the PANI/NiO/TiO_2_ sensor exhibited rapid response behavior to 1 ppm-50 ppm acetone, with a response of 11.9 to 50 ppm acetone, a limit of detection of 176.2 ppb, response/recovery times of 150/290 s, and linearity across 1–50 ppm with $R^{2}=0.986$. Cross-responses to CO and benzene were below 2%, and the six-month drift was under 5%. At 50%RH, proton-exchange in PANI increased conductivity and reduced the response to 6.2, indicating the weak humidity-resistance. 

**Figure 5:**
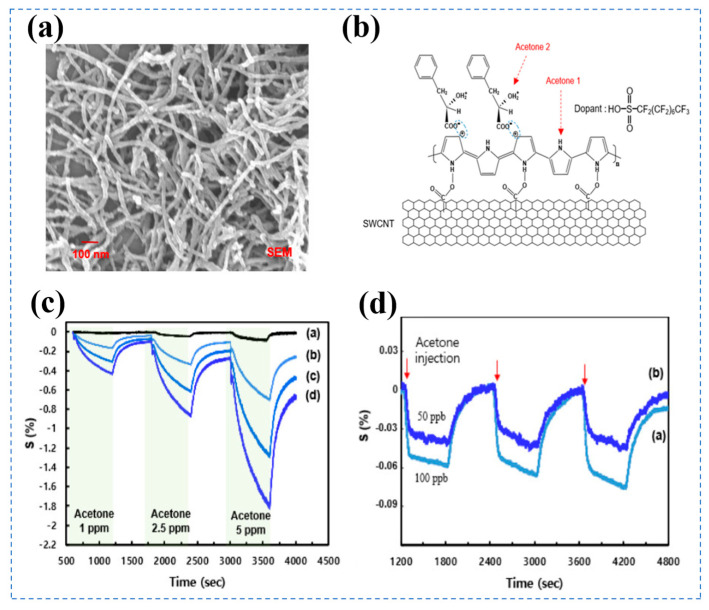
(**a**) The scanning electron microscopy (SEM). (**b**) Schematic of and the chemical structure of C8F-doped-PPy/PLA@SWCNT core-shell-shaped nanocomposites for C_3_H_6_O gas sensing. (**c**) Continuous dynamic responses of C8F-doped-PPy/PLA@SWCNT sensors with different PLA molar ratios (0, 0.1, 0.3, 0.5) to 1–5 ppm acetone (C_3_H_6_O) at 25 °C and 0%RH (dry air). (**d**) Response characteristics of the sensor with PLA_0.5_ molar ratio to 50–100 ppb acetone (C_3_H_6_O) under the same conditions, reprinted with permission from Ref. [108]. Copyright 2025, MDPI.

Byeon et al. synthesized C8F-doped PPy/PLA@SWCNT core-shell nanocomposites (40 nm in diameter and 1–5 µm in length) via emulsion polymerization, which were drop-coated onto Au electrodes to form sensing layers for constructing acetone gas sensors [108]. Figure 5a shows the fibrous morphology with uniform dimensions of the composites. The structural schematic of the core-shell system is exhibited in Figure 5b, clarifying the binding mode between C8F-doped PPy/PLA and SWCNT, as well as the dopant’s action sites. Figure 5c presents the dynamic response curves of the sensor to 1 ppm, 2.5 ppm, and 5 ppm acetone. Additionally, the sensor shows excellent repeatability to trace acetone (Figure 5d). At room temperature, the sensor exhibits an effective response to 50 ppb acetone, with the response value increasing with concentration in the range of 1–5 ppm, and maintains stable signals within 0–80% RH. It enables noninvasive detection of acetone in exhaled breath (a biomarker associated with diabetes and fat metabolism). The sensor does not specify the response and recovery time, with the limitation that no effective detection signal can be obtained for 1 ppm acetone at RH above 40%. Adhikari et al. designed polypyrrole nanotubes (PPNT)—based gas sensors to enable trace acetone detection [109] under ambient conditions, adopting a soft-template-assisted Taguchi-type thick-film fabrication approach. The sensor exhibited a high response of 47% to 0.5 ppm acetone, a low LOD of 500 ppb, and short response/recovery times of 5.4 s and 73.9 s, respectively. The underlying acetone sensing mechanism relies on charge transfer interactions between acetone molecules and the polypyrrole backbone. The device worked effectively over an acetone concentration range of 0.5–25 ppm and exhibited strong selectivity against ethanol and ammonia interference. This selectivity is attributed to the formation of hydrogen bonds between the C=O groups of acetone and the N-H groups of pyrrole. 

Davis et al. further advanced flexible acetone sensing by developing a paper-based device that utilized a zinc oxide–polyaniline composite as the sensing layer and a carbon-black/polyacrylonitrile composite as the electrode material [110]. The sensor was fabricated on a 70 GSM paper substrate using a combination of doctor-blading and screen-printing techniques. The device demonstrated a high sensitivity of 0.02/100 ppm, with rapid response/recovery times of 4/15 s at room temperature. In addition, the sensor exhibited reliable detection of acetone concentrations between 260 and 1000 ppm and maintained stable electrical performance after one thousand bending cycles, confirming its mechanical robustness. 

To facilitate a clear and systematic comparison of the gas-sensing performances discussed above, Table 2 comprehensively summarizes the key parameters of recent reports. The conducting polymer-based gas sensors exhibited a high response and low LOD for the biomarker gases, holding potential for real-time detection of exhaled breath biomarkers. Moreover, Table 2 presents the effect of material modification strategies (S_F_/S_N_), which provide effective methods to further enhance the gas-sensing performance of polymer-based gas sensors.

As summarized in Table 2, the detection limits of certain sensors fall below the clinically relevant concentration ranges. To meet the stringent detection limit requirements for clinical diagnostics, researchers have developed a series of high-performance sensing systems tailored for different target gases. These systems exhibit detection limits consistently lower than the clinically relevant thresholds, making them well-suited for practical clinical applications, such as MXene@Zn_3_In_2_S_6_@PANI for NH_3_ [70], SnO_2_/rGO)/PANI for H_2_S [83], PANI sensor for H_2_ [92], ZnO/PANI sensor for NO_2_ [101], and PANI/NiO/TiO_2_ composite sensor for acetone [112]. The aforementioned sensors, through rational material structure design and synthesis process optimization, provide reliable technical support for the accurate detection of various gases in clinical scenarios. However, the detection limits of some other sensors still do not meet clinical requirements, though they demonstrate promising performance in other aspects, such as response/recovery time and detection range. Subsequent optimization and improvement of these sensors are therefore necessary.

Moreover, the three representative conducting polymers discussed in this chapter (PANI, PPy, and PTh), exhibit pronounced differences in molecular structure, doping behavior, charge-transport characteristics, and environmental stability, which fundamentally determine their suitability for detecting breath-borne biomarkers. PANI, benefiting from its reversible protonation/deprotonation transitions and multiple accessible oxidation states, shows strong chemical affinity toward alkaline or amine-containing gases such as NH_3_. These features make PANI particularly advantageous for diagnosing diseases associated with elevated basic analytes. However, its intrinsically hydrophilic backbone renders the material susceptible to humidity fluctuations, potentially introducing additional noise in real breath environments. PPy, characterized by a relatively rigid conjugated backbone and efficient π–π stacking, enables rapid charge transport and exhibits pronounced dedoping behavior in the presence of strong oxidizing gases such as NO_2_, resulting in fast, distinct, and highly reproducible resistance changes. Nevertheless, PPy is prone to structural degradation under harsh oxidative conditions, which may limit its long-term stability. In contrast, PTh and its derivatives possess inherently hydrophobic backbones, enhanced chemical robustness, and reduced baseline drift, allowing them to maintain superior environmental stability in humid or compositionally complex breath matrices. These properties render PTh particularly suitable for detecting low-polarity VOCs such as acetone, although its intrinsic reactivity toward highly polar or strongly electron-withdrawing gases is comparatively weaker and typically requires composite engineering or functionalization strategies to improve sensitivity.

## 4. Emerging Trends in Intelligent Polymer Gas Sensors and Electronic Noses

In the preceding three chapters, the material systems, sensing mechanisms, and performance-enhancement strategies of conducting-polymer gas sensors were comprehensively reviewed, forming a solid foundation for the subsequent development. As elaborated earlier, conducting polymer-based gas sensors hold exceptional promise for noninvasive disease diagnosis via exhaled biomarkers, owing to their low operating temperature, facile chemical functionalization, and mechanical flexibility. However, single conducting-polymer sensors are still plagued by significant drawbacks—most notably, poor selectivity among structurally similar analytes, and high susceptibility to humidity interference, which often leads to signal drift and reduced reliability. To date, many efforts for performance improvement have focused purely on the sensing material itself—such as doping strategies, nanostructuring, or creating composites. While such material-level optimizations have delivered substantial gains in sensitivity or response speed, they alone cannot fully overcome the challenges posed by realistic sensing scenarios. In actual applications, one must often contend with multi-component gas mixtures, fluctuating concentrations, and dynamically changing environmental conditions. Under such complex conditions, material design alone is insufficient to guarantee the high accuracy, real-time discrimination, and long-term stability required for practical deployment.

Therefore, in addition to material improvement, intelligent data processing is becoming critical. Effective interpretation, compensation, and fusion of multidimensional sensor signals are now essential. In this context, machine learning has emerged as a particularly powerful tool: algorithms for pattern recognition, nonlinear feature extraction, drift correction, dimensionality reduction, and adaptive optimization can significantly enhance system performance. Recent review studies have demonstrated how advanced machine learning techniques can greatly improve the stability, selectivity, and robustness of electronic-nose (E-nose) systems based on gas sensor arrays [118,119]. Thus, machine learning has become increasingly central to the development of high-performance, multifunctional E-nose platforms that fully exploit the advantages of conducting-polymer sensing materials.

### 4.1. Applications of Machine Learning in Polymer-Based Gas Sensors

The rapid advancement of gas-sensing technology has highlighted several persistent and fundamental challenges for conventional polymer-based gas sensors, particularly in terms of sensitivity, selectivity, and the reliable detection of low-concentration analytes. These limitations become even more pronounced under realistic conditions involving complex gas mixtures, fluctuating environmental parameters, and inevitable sensor aging or drift. Traditional signal-processing approaches—typically based on linear fitting, simple filtering, or low-dimensional statistical analysis—often fail to accommodate the inherently nonlinear response behaviors of polymer sensors, their susceptibility to multi-factor interference, and the gradual degradation of sensing performance over extended operation. These constraints significantly hinder the deployment of polymer-based gas sensors in high-precision or long-term monitoring applications. 

In recent years, machine learning (ML) has emerged as a powerful and versatile strategy for overcoming these obstacles. By leveraging data-driven modeling and adaptive computation, ML can intelligently analyze multidimensional sensor output signals, enabling robust pattern recognition, effective feature extraction, drift correction, and real-time decision making. These capabilities substantially improve detection accuracy, sensitivity, selectivity, and environmental robustness, thereby pushing polymer-based gas sensors closer to practical, application-ready systems [6,13,120].

In polymer-based sensing systems, machine learning (ML) techniques are predominantly applied to advanced signal processing and pattern recognition. The responses of polymer sensors are often influenced by multiple environmental and operational factors—such as temperature fluctuations, humidity variations, background gases, and intrinsic drift—resulting in high-dimensional, nonlinear, and temporally complex signal profiles. Extracting reliable and discriminative features from such data poses significant challenges for traditional analytical methods, which typically rely on predefined transformations or linear assumptions and thus struggle to capture essential variations embedded in the sensor outputs.

ML algorithms provide a powerful alternative by enabling data-driven modeling of complex feature spaces. Methods such as support vector machines (SVMs), random forests (RF), principal component analysis (PCA), and convolutional neural networks (CNNs) can efficiently handle nonlinear relationships, identify latent structures, and enhance the interpretability of multidimensional sensing data [121]. These approaches substantially improve the accuracy of gas classification, concentration prediction, and interference compensation, offering a more robust and adaptive framework compared with conventional signal-processing techniques [17]. 

During prolonged operation, polymer gas sensors are inherently susceptible to performance degradation caused by material aging, humidity and temperature variations, and device-to-device inconsistencies, which collectively lead to pronounced temporal drift. To address this long-standing challenge, Zhang et al. proposed an unsupervised attention-based multi-source domain adaptation framework (AMDS-PFFA) and systematically validated its performance on the widely used UCSD Gas Sensor Drift Dataset [122]. The results demonstrated that the proposed method achieved stable feature alignment across different time batches and sensor sources, yielding an average classification accuracy of 83.2% on the public dataset and further improving to 93.9% on a self-collected sensor dataset. These findings indicated that incorporating attention mechanisms into latent feature spaces to decouple intrinsic gas-related features from time-dependent drift components can effectively suppress response drift, thereby significantly improving the long-term stability of polymer-based gas-sensing systems.

Reproducibility remains a critical bottleneck for polymer-based gas-sensing systems, even when advanced ML algorithms are employed to enhance data interpretation. The intrinsic variability introduced during polymer synthesis, such as fluctuations in monomer oxidation level, heterogeneous microstructures, film-thickness inconsistencies, and dopant instability, often leads to device-dependent response patterns. As a result, an ML model trained on one sensor or batch may exhibit substantial performance degradation when applied to different fabrication batches or sensor units, thereby limiting cross-device generalizability and practical deployment. To overcome these challenges, robust calibration strategies are indispensable for ensuring stable performance across heterogeneous sensor populations. A representative example is the calibration-transfer framework developed by Fonollosa et al., who investigated a large array of polymer-coated chemoresistive sensors fabricated across multiple batches. By applying direct standardization, multi-sensor baseline alignment, and drift-aware normalization, their model successfully reduced inter-device variability by more than 70%, enabling normalization. Their model successfully reduced inter-device variability by more than 70%, enabling reliable cross-device prediction without the need for retraining the entire model [123]. This work demonstrates how ML-assisted calibration transfer can compensate for fabrication-induced discrepancies and significantly improve reproducibility in practical deployments.

A promising alternative to cross-device calibration for long-term deployment lies in the development of self-calibrating or calibration-free sensor architectures. Vergara et al. applied a manifold-learning-based correction model to the UCSD chemical sensor drift dataset, enabling automatic alignment of temporal drift trajectories across multiple years. Their approach reduced calibration effort by more than 50% while restoring year-to-year classification accuracy that had degraded due to material aging and environmental fluctuations [124]. Moreover, recent work integrating adaptive normalization into recurrent neural network (RNN) models demonstrated the ability to maintain stable prediction outputs over extended operational periods, even under humidity-induced swelling or temperature-dependent structural changes in polymer films.

Despite advances in drift compensation, the reliable detection of trace-level gases remains a critical challenge in polymer-based gas sensing. At low concentrations, sensor responses are typically characterized by low signal-to-noise ratios and are highly susceptible to humidity variations and background gas interference, rendering conventional signal-processing methods insufficient for robust quantitative analysis. Recent studies have shown that deep neural networks integrated with signal modeling and drift-compensation strategies can substantially enhance detection performance under such adverse conditions. For instance, ML-assisted frameworks combining spectral–temporal neural architectures with lightweight learning strategies have demonstrated stable detection of ethylene at concentrations as low as 1 ppm [125], even in the presence of high humidity and CO_2_ background interference. These results highlight the strong potential of ML-based approaches for extending the detection limits of polymer gas sensors in complex real-world environments.

Collectively, these studies illustrate that reproducibility in polymer-based gas-sensing systems depends not only on stable material design but also on advanced ML-enabled calibration strategies. Approaches such as calibration transfer, domain alignment, drift-aware normalization, and self-calibrating algorithms are essential for bridging the performance gap between controlled laboratory conditions and real-world, long-term operation.

Machine learning plays a pivotal role in polymer-based sensor systems, extending its utility beyond classification and quantification to encompass adaptive control and real-time optimization processes. Deep learning models can dynamically adjust sensor operating parameters in response to environmental variations, maintaining optimal performance [126]. Zhou et al. proposed a recurrent neural network (RNN)-based model that compensates for sensor drift in real time, ensuring long-term stability [119]. Additionally, reinforcement learning (RL) approaches have been applied to sensor arrays for self-calibration and online adaptation, enabling consistent high recognition accuracy and operational reliability over extended periods [127]. 

Machine learning has also proven effective in enhancing multi-gas discrimination capabilities in conductive polymer–based gas-sensing systems. Conductive polymers, such as polyaniline (PANI), are widely employed in resistive gas sensors due to their tunable electrical conductivity, facile functionalization, and pronounced responses to a broad range of gas molecules. Representative studies have constructed sensor arrays based on multiple PANI-derived nanocomposites and applied ML-based pattern recognition methods, such as principal component analysis combined with probabilistic classifiers, to achieve multi-gas discrimination [128]. Under controlled experimental conditions, these systems have demonstrated gas-classification accuracies approaching 99%. Although such performance is typically obtained under relatively stable environments and single-gas exposure scenarios, these studies nevertheless provide compelling evidence that ML techniques can effectively exploit the latent discriminative information embedded in conductive polymer sensor arrays.

Overall, machine learning techniques not only markedly enhance individual performance metrics of polymer-based gas sensors, including sensitivity, selectivity, and operational stability, but also fundamentally drive a paradigm shift from conventional passive signal detection toward intelligent and adaptive sensing systems. By integrating multimodal sensing data with advanced deep learning frameworks, such as domain adaptation, attention mechanisms, and task-coupled feature learning, polymer-based gas-sensing systems achieve coordinated optimization across gas identification, concentration estimation, sensor-drift suppression, and environmental adaptability. Consequently, these systems are capable of delivering not only high-accuracy and robust gas detection but also advanced functionalities such as trend prediction, anomaly detection, and long-term autonomous operation. Collectively, these advances establish a solid foundation for the development of highly reliable, continuously operable, and intelligent polymer-based gas-sensing platforms tailored for long-term monitoring in complex and dynamic environments [129].

### 4.2. Design and Future Trends of Electronic-Nose (E-Nose) Sensor Arrays

Electronic noses (E-noses) emulate the human olfactory system to identify and quantify complex gas mixtures. Polymer-based gas sensors serve as essential building blocks in these systems owing to their high sensitivity, low cost, and versatile tunability in chemical structure and surface functionality [130,131]. With the rapid development of artificial intelligence (AI) and Internet of Things (IoT) technologies, E-noses are increasingly moving toward higher levels of integration, intelligence, and miniaturization, enabling more flexible, efficient, and robust gas-sensing capabilities across diverse application scenarios [132].

The overall performance of an E-nose is largely determined by the design of the sensor array and the strategies used for multidimensional signal fusion. Each sensor in the array exhibits partially overlapping, yet distinct responses to different analytes, and the use of advanced fusion algorithms—coupled with machine learning (ML)-based feature extraction—greatly improves the system’s ability to discriminate, classify, and quantify complex gas mixtures with high precision [133]. Meanwhile, functionalization of polymer sensing materials, such as through metal-ion doping or incorporation of selective functional groups, enhances their interaction strength with target molecules and improves stability under high-humidity or variable environmental conditions [134].

Recent advancements in micro-electromechanical systems (MEMS) have further enabled the development of miniaturized and even wearable polymer-based sensor arrays, featuring low power consumption and rapid response/recovery characteristics. When integrated with adaptive ML algorithms, these miniaturized systems can achieve self-learning, environmental compensation, and reliable multidimensional data processing, thereby maintaining stable performance in dynamic sensing environments [132]. Looking forward, next-generation E-nose systems are expected to incorporate autonomous “perception–learning–decision” loops, enabling real-time environmental interpretation and intelligent optimization. With continued progress in materials engineering, device integration, and intelligent algorithms, polymer-based E-noses will play an increasingly significant role in environmental monitoring, food quality control, industrial safety, and medical diagnostics.

### 4.3. Future Perspectives and Challenges

Despite substantial advancements, polymer-based gas sensors and E-nose systems still encounter several enduring challenges that hinder their broader practical deployment. One of the most critical issues is long-term stability and environmental robustness. Variations in humidity, temperature fluctuations, and progressive sensor aging can introduce pronounced signal drift, baseline instability, and degradation of sensing sensitivity, ultimately compromising detection reliability over extended operation periods [119]. 

Humidity interference constitutes one of the most persistent and technically challenging environmental factors affecting the performance of conducting polymer–based gas sensors, particularly in high-humidity scenarios such as exhaled breath analysis. The underlying mechanisms arise from multiscale interactions between water molecules and the sensing material across adsorption, diffusion, and charge-transport domains. Water vapor readily interacts with polar functional groups on conducting-polymer backbones and the surfaces of composite fillers, occupying critical adsorption sites and altering the local surface energy landscape [135]. This competitive adsorption diminishes the ability of target analytes to engage in charge-transfer or doping–dedoping reactions [136], thereby reducing sensitivity and disrupting selectivity. Under high relative humidity, water may further assemble into multilayer adsorption films that hinder gas diffusion into the active sensing region. Concurrently, penetrating water molecules induce polymer swelling within amorphous domains, plasticizing chain segments, enlarging interchain spacing, and disturbing π–π stacking interactions [13]. Such microstructural rearrangements impair charge-transport continuity and introduce baseline drift and increased noise; in hydrophilic polymers, they may even lead to dopant migration or partial dissolution of oligomeric segments, resulting in irreversible morphological deterioration. Water also perturbs the local chemical environment, shifting doping equilibria, such as protonation in PANI or localized redox states in PPy, and generating nonlinear humidity-dependent conductivity variations. To mitigate these intertwined effects, both materials engineering and system-level strategies are essential. Hydrophobic surface modification, engineered inorganic–organic heterostructures with controlled surface energy, backbone rigidification or crosslinking to suppress swelling, as well as real-time humidity monitoring combined with machine-learning-based signal decoupling and drift correction, collectively provide robust pathways toward achieving reliable sensing under high-humidity conditions.

Temperature fluctuations represent an equally critical source of interference, owing to the strong temperature dependence of both intrinsic charge-transport mechanisms and gas adsorption/desorption kinetics in conducting polymers. Charge transport in these materials is typically governed by thermally activated hopping processes, such that elevated temperatures lower activation barriers, enhance carrier mobility, and shift doping equilibria, leading to measurable baseline resistance drift even in the absence of analytes [137]. At the same time, adsorption isotherms and kinetic parameters exhibit Arrhenius-type behavior, whereby increasing temperature decreases equilibrium adsorption capacity and accelerates desorption, resulting in reduced steady-state response magnitudes but faster transient dynamics, as evidenced in polymer-coated sensor arrays where both humidity and temperature modulate sensitivity patterns [138,139]. Temperature and humidity effects frequently co-occur and interact, as temperature modulates water vapor pressure, polymer hydration behavior, and swelling kinetics, giving rise to complex multivariable environmental interference that cannot be addressed solely through material design. Therefore, precise temperature-compensation strategies are indispensable. Co-integration of high-accuracy temperature sensors with the sensing element, development of real-time calibration models, and implementation of machine-learning frameworks trained across multidimensional temperature–humidity–concentration spaces can effectively correct baseline offsets and response deviations. Meanwhile, materials-level approaches, such as enhancing backbone rigidity, stabilizing dopant chemistry, or incorporating thermally conductive fillers to homogenize local thermal distribution, can reduce intrinsic thermal sensitivity. Together, addressing humidity and temperature interference through coordinated material optimization and advanced signal compensation forms a crucial foundation for achieving stable, reliable, and environmentally robust performance in conducting-polymer gas-sensing platforms.

To address these limitations, future research should place greater emphasis on optimizing polymer chain structures, tailoring intermolecular interactions, and engineering stable interfacial chemical reactions. Furthermore, the incorporation of adaptive self-calibration and drift-compensation algorithms will be indispensable for improving long-term reproducibility and ensuring consistent sensor performance in dynamic environments. In addition to stability, scalability and fabrication consistency remain major bottlenecks for industrialization. Polymer-based sensing films often require complex synthesis routes or multi-step functionalization, which increases production cost and leads to batch-to-batch variability that undermines quality control. Therefore, the development of low-cost, environmentally friendly, and highly scalable fabrication strategies—such as solution-processable printing, large-area coating, or roll-to-roll manufacturing—is essential for enabling uniform mass production and accelerating commercial translation [131].

With the rapid convergence of IoT and AI technologies, gas-sensing architectures are evolving toward highly intelligent, interconnected, and edge-computing-enhanced systems. Next-generation polymer-based gas sensors are expected not only to provide high sensitivity and selectivity but also to support autonomous functions such as real-time self-calibration, remote monitoring, multi-node data fusion, and on-device decision making. Such capabilities will be crucial for building scalable, energy-efficient, and truly smart sensing networks capable of operating reliably in complex real-world environments [133].

In conclusion, the synergistic integration of advanced polymer materials, intelligent data-processing frameworks, and high-performance electronic engineering is poised to drive transformative progress in polymer-based gas sensors and E-nose systems. This interdisciplinary convergence will enable the development of autonomous, robust, and intelligent sensing platforms with promising applications in environmental monitoring, medical diagnostics, industrial safety, and smart-home ecosystems.

## Figures and Tables

**Table 1:** Potential biomarkers for disease diagnosis based on exhaled breath.

Gas Species	Disease/Condition Associated with the Biomarker	Reported Concentration Range	Ref.
NH_3_	Chronic kidney disease	153–130,000 ppb	[63]
NH_3_	Heliobacter pylori Infection	50–400 ppb	[63]
H_2_S	Halitosis	0.1–0.5 ppm	[64]
H_2_	Small intestinal bacterial overgrowth	an increase of ≥20 ppm	[63]
NO_X_	Asthma	FeNO levels ≥40 ppb	[65]
Acetone	Type 1 diabetes mellitus	2.2–22 ppm.	[66]
Acetone	Type 2 diabetes mellitus	1.76–9 ppm	[66]
Acetone	Diabetes mellitus	>1.8 ppm.	[66]

**Table 2:** Gas-sensing performance of polymer-based and hybrid composite materials.

Sensing Material	Target Gas	R	LOD	Tres/Trec	SF/SN	Ref.
MXene@Zn_3_In_2_S_6_@PANI	NH_3_ (100 ppm)	484.28% ^b^	573.105 ppb	200.2 s/95.7 s	2.39	[70]
PVA/CS/PANI	NH_3_ (100 ppm)	79.49% ^b^	571 ppb	150 s/18 s	2.41	[71]
PANI-CeO_2_	NH_3_ (50 ppm)	262.7% ^b^	16 ppb	-/-	1.58	[68]
PPy-coated TPU	NH_3_ (100 ppm)	1.2 ^a^	-	23 s/43 s	1.30	[72]
ZnO/PANI nanocomposites	NH_3_ (500 ppm)	5990 ^a^	0.1 ppm	-/-	5990.00	[73]
PANI/LIG	NH_3_ (60 ppm)	68% ^b^	5 ppm	53 s/167 s	6.80	[111]
s-PANI@PDMS	NH_3_ (60 ppm)	4.02 ^a^	0.5 ppm	43 s/53 s	1.24	[74]
PANI@Cu_2_@MWCNT_2_	NH_3_ (100 ppm)	43% ^b^	100 ppm	10 s/13 s	2.39	[112]
urchin-like polypyrrole	NH_3_ (10 ppm)	3% ^b^	0.01 ppm	1 s/30 s	10	[75]
PTh/SWCNT nanocomposite	NH_3_ (2000 ppm)	27% ^b^	5 ppm	60 s/60 s	12.6	[76]
PAni-SnCl_2_-PEDOT:PSS	H_2_S (1 ppm)	0.8% ^b^	28 ppb	70 s/-	-	[81]
SnO_2_/rGO/PANI	H_2_S (5 ppm)	76.25% ^b^	50 ppb	80 s/88 s	12.0	[82]
PT-WO_3_	H_2_S (100 ppm)	13.3 ^a^	<2 ppm	-/-	5.32	[83]
PANI/GOA	H_2_S (50 ppm)	49% ^b^	1 ppm	1 s/135 s	12.25	[85]
PANI/SnO_2_/rGO	H_2_S (100 ppm)	56% ^a^	-	35 s/40 s	4.67	[86]
ZnMn_2_O_4_/PPy NC	H_2_ (2500 ppm)	1.02 ^a^	2500 ppm	21.6 s/97.2 s	1.2	[113]
PTh/g-C_3_N_4_ NC	H_2_ (10,000 ppm)	29.3% ^b^	1000 ppm	69 s/83 s	5.77	[114]
CSA-Doped PANI NFs	H_2_ (10,000 ppm)	3% ^b^	10,000 ppm	-	10	[93]
PANI/SnO_2_/Pd NC	H_2_ (400 ppm)	546.14% ^b^	50 ppm	547 s/164 s	40	[115]
PANI hollow nanotubes	H_2_ (1 ppm)	29% ^b^	1 ppm	15 s/17 s	4.83	[91]
PMMA/Pd NP/SLG hybrid	H_2_ (20,000 ppm)	66.37% ^b^	250 ppm	64.2 s/676.8 s	1.33	[116]
PANI/Ag_2_O/GO	NO_2_ (25 ppm)	5.85 ^b^	-	100 s/140 s	2.34	[96]
ZnO/PANI nanoflake arrays	NO_2_ (10 ppm)	28 ^a^	10 ppb	32 s/18 s	23.53	[97]
TPMNTs	NO_2_ (1 ppm)	11.96 ^b^	0.12 ppb	9 s/11 s	5.98	[98]
PANI-PSSA	NO_2_ (20 ppm)	0.2 ^a^	-	8 s/120 s	1.2	[99]
INPTh	NO_2_ (100 ppm)	47.58% ^b^	1 ppm	-	6.93	[100]
SWCNT TFTs	NO_2_ (0.5 ppm)	0.928 ^b^	0.069 ppm	8 s/8 s	2.72	[117]
polythiophene, PTh	NO_2_ (10 ppm)	9% ^b^	10 ppm	220 s/585 s	-	[101]
PANI/NiO-loaded TiO_2_ NPs	acetone (50 ppm)	11.3 ^a^	176.2 ppb	150 s/290s	5.5	[107]
C_8_F-doped-PPy/PLA@SWCNT	acetone (5 ppm)	1.45 ^b^	50 ppb	-	72.5	[108]
PPNT	acetone (0.5 ppm)	47% ^b^	500 ppb	5.4 s/73.94 s	235	[109]
ZnO-PANI composite on paper	acetone (100 ppm)	0.02 ^b^	260 ppm	4 s/15 s	4	[110]

^a^ R = Ra/Rg or Rg/Ra, ^b^ R = (Ra−Rg)/Ra × 100% or (Rg−Ra)/Rg × 100%.

## Data Availability

Dataset available on request from the authors.
